# Transgastric repair of transfixing gastroesophageal junction gunshot wound: video case report

**DOI:** 10.1093/jscr/rjab160

**Published:** 2021-05-21

**Authors:** Javier Vela, Caterina Contreras, Julián Varas, Pablo Ottolino, Juan Pablo Ramos, Gabriel Escalona, Alfonso Diaz, Pablo Achurra, Marco Ceroni

**Affiliations:** Surgery Department, Pontificia Universidad Católica de Chile, Santiago, Chile; Surgery Department, Complejo Asistencial Dr. Sótero del Río, Santiago, Chile; Surgery Department, Pontificia Universidad Católica de Chile, Santiago, Chile; Surgery Department, Complejo Asistencial Dr. Sótero del Río, Santiago, Chile; Surgery Department, Pontificia Universidad Católica de Chile, Santiago, Chile; Surgery Department, Complejo Asistencial Dr. Sótero del Río, Santiago, Chile; Surgery Department, Complejo Asistencial Dr. Sótero del Río, Santiago, Chile; Surgery Department, Complejo Asistencial Dr. Sótero del Río, Santiago, Chile; Surgery Department, Complejo Asistencial Dr. Sótero del Río, Santiago, Chile; Surgery Department, Complejo Asistencial Dr. Sótero del Río, Santiago, Chile; Surgery Department, Pontificia Universidad Católica de Chile, Santiago, Chile; Surgery Department, Complejo Asistencial Dr. Sótero del Río, Santiago, Chile; Surgery Department, Complejo Asistencial Dr. Sótero del Río, Santiago, Chile

## Abstract

Managing traumatic injuries of the gastroesophageal junction (GEJ) is infrequent due to associated lesions of adjacent highly vascularized organs. Its anatomical localization in the upper abdomen makes the repair challenging to perform. A stable 23-year-old male was presented at the emergency department with two thorax gunshot wounds. Computed tomography revealed air in the periesophageal space and right hemopneumothorax with no injury of the major vessels. A chest tube was placed and the patient was transferred hemodynamically stable to the operating. Abdominal exploration identified injuries to the left diaphragm; liver lateral segment; 1-cm transfixing perforation of the GEJ and right diaphragmatic pillar. Primary repair of the GEJ was performed and patched with a partial fundoplication. The diaphragm was repaired and the liver bleeding controlled. Finally, drains and a feeding jejunostomy were placed. The patient had an uneventful early postoperative course and was discharged home on the 12th postoperative day.

## INTRODUCTION

Injuries of the gastroesophageal junction (GEJ) are infrequent due to associated trauma of adjacent highly vascularized organs such as the heart, liver and major vessels [1, 2]. On-site mortality is often high, with little chances to transfer the patient to the emergency department. Moreover, the GEJ is an anatomical region hard to reach in surgery, due to its localization in the upper portion of the abdomen and often requires liver mobilization or the division of short gastric vessels to provide adequate exposure [3]. Computed tomography (CT) is usually contraindicated because of hemodynamic instability, so most decisions have to be taken intraoperatively.

Surgical management of these injuries has been poorly defined. Textbooks and case reports describe multiple options ranging from simple primary repair to highly complex alternative such as esophageal transection, total gastrectomy or damage control procedures. In trauma surgery, we usually prefer simple methods of repair. This report aims to present the surgical case of a patient with transfixing GEJ gunshot wound and primary repair through an anterior approach. A video of the procedure and a literature review are provided (see Supplementary material).

## CASE REPORT

**Figure 1:**
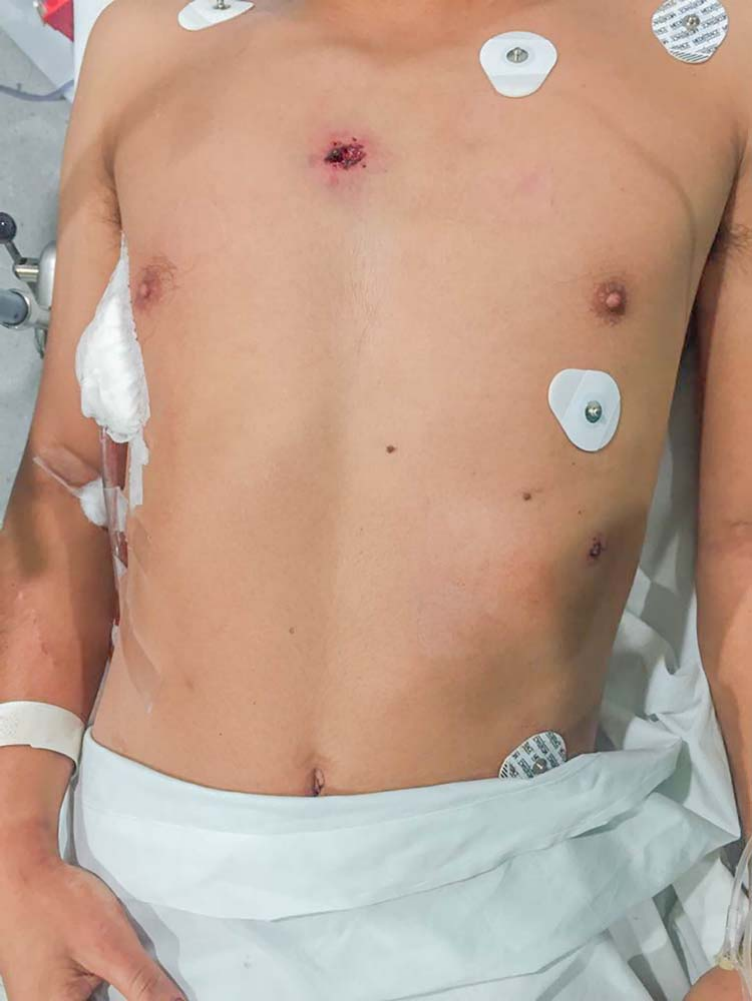
Patient upon arrival; two gunshot wounds are visible at the anterior and left thorax.

A 23-years-old male presented at the emergency department with a gunshot wound to the left thoracoabdominal region. After advanced trauma life support (ATLS)-based management, exposure revealed two gunshot wounds on the anterior and left lateral thorax (Fig. 1). The patient condition was stable so a contrast-enhanced CT was performed, revealing air in the periesophageal space, right hemopneumothorax, hepatic subcapsular hematoma and a mild pneumoperitoneum. A pleurostomy was installed and 400 cc of blood were drained. In the operating room an exploratory midline laparotomy was performed identifying a diagonal bullet trajectory that compromised from anterior-left to posterior-right: (i) left diaphragm; (ii) liver lateral segment; (iii) 1-cm transfixing perforation of the anterior gastric fundus; (iv) GEJ posterior wall and (v) right diaphragmatic pillar. The Aorta and inferior vena cava presented no injuries.

**Figure 2:**
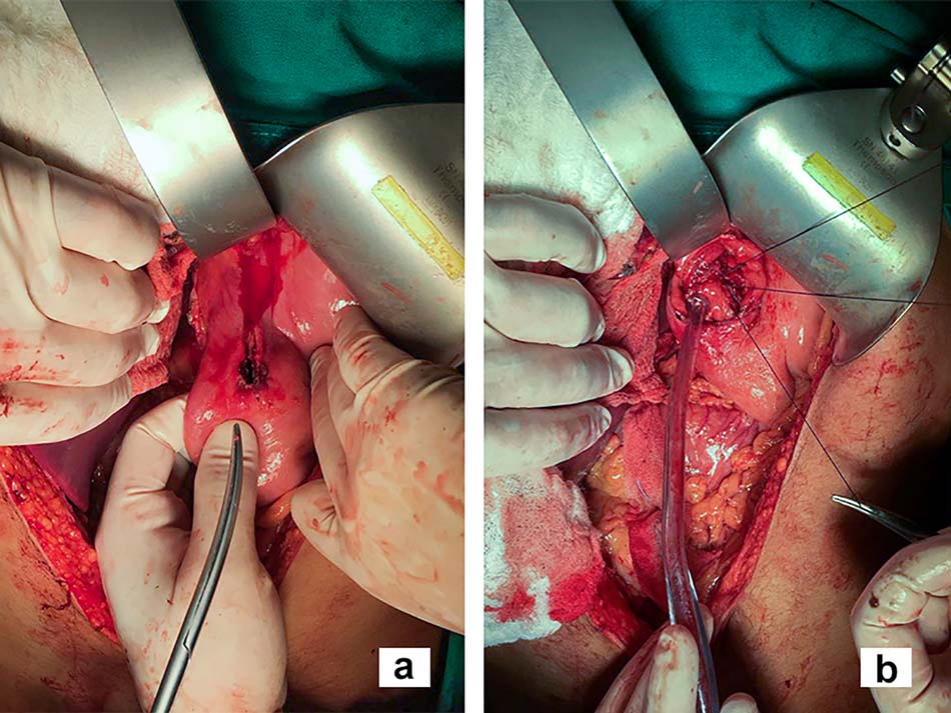
Transgastric approach; in the image, the closure of the GEJ injury is done through an anterior gastrotomy (**b**) made by expanding the perforating lesion in the stomach anterior wall (**a**).

The liver left lateral lobe was mobilized and retracted medially. To provide adequate exposure of the GEJ posterior wall without complete mobilization of the stomach and esophagus, an anterior transgastric approach through the anterior wall penetrating injury was performed (Fig. 2). Primary repair of GEJ posterior wall was done with a 2-0 Vicryl® interrupted stiches. After wound edge debridement, anterior gastric wall was repaired with 2-0 Vicryl® and reinforced with partial fundoplication. Repair of the diaphragm and hemostatic control of the lateral segment of the liver were conducted. Finally, drains and a feeding jejunostomy were placed. On the third day postoperatively, a contrasted image of the repair was obtained without evidence of a leak, so the patient was progressively fed per mouth. A low debit biliary fistula from the liver's injured lateral segment developed and was treated conservatively through the drainage, until it was successfully removed. The patient was discharged home on the 12th postoperative day.

## DISCUSSION

Traumatic injuries of the GEJ are infrequent at the emergency department. A series of 28 cases, reported that the main etiology were penetrating traumas (96%), whether by gunshot (81%) or by stabbing (19%) [4]. The mortality reported was high (25%), mainly due to hemodynamic instability of the patient.

In order to achieve the GEJ exposure, transection of the triangular ligament and mobilization of the lateral segment of the liver left lobe is often described [5]. For exposure of posterior injuries, full mobilization of the distal esophagus and proximal stomach is needed. We present an alternative procedure with an anterior approach with minimal mobilization of the GEJ. Enlarging the anterior gastric wall injury provided excellent exposure for primary repair of the posterior wall. According to the intraoperative findings, a feeding jejunostomy or a postpyloric nasogastric tube may be installed for early enteral feeding and used in case of leak or stricture.

Repair techniques for GEJ injury vary along the literature describing a wide range of procedures such as total gastrectomy, esophageal transection, wedge resection, esophagogastrectomy, T-tube placement, intentional fistulization or simple primary repair. Some of these techniques are highly complex and involve potentially morbid outcomes, including postoperative leak and stricture. The only and largest series of GEJ traumatic injuries published to date by Schellenberg *et al.* describe 28 cases and summarize the procedures accomplished in a single US center. Of these, 21 patients undergo primary sutured repair either on a one-layer fashion (*n* = 8) or a two-layer (*n* = 13). Other patients followed a damage control approach and had the GEJ injury stapled off for delayed reconstruction (*n* = 4) [4]. Textbooks states that lesions of the GEJ smaller than 2 cm can be treated with a primary repair with a 2-0 Vicryl® interrupted suture in a one or two-layer fashion or with a 3-0 silk Lembert suture [5].

The surgical approach can be performed by laparotomy, thoracotomy or thoracoabdominal incision and is decided upon the injury location or trayectory and the suspected associated injuries [6]. In the same series, most GEJ injury was approached by laparotomy, and only one case required a thoracotomy to proceed with the repair [4]. In light of this finding, the authors suggest to approach these injuries by a laparotomy as a first step and perform a thoracotomy when needed. Associated injuries are frequent, and ~96% of these patients present one. The more frequent were liver (57%), diaphragm (43%) and spleen (32%) [4]. The diaphragmatic injury enables the flow of gastric content to the pleural cavity, so this space must be carefully washed. In most cases, after the gastric perforation closure, the clearing of the pleural space can be done through the diaphragmatic lesion. In other scenarios, an enlargement of the initial injury can be a suitable option to ensure the evacuation of the pleural contamination. The closure of the diaphragm is always performed and a chest tube may be placed [5]. Most minor hepatic injuries can be successfully treated conservatively, but caution should be applied because, in the presence of untreated diaphragmatic lesions, a biliopleural fistula can develop [3]. Fundoplication is used to reinforce the injuries of the distal esophagus, and a drainage is strongly advised to be installed [6].

In conclusion, GEJ injuries are rare and multiple management options have been described. We present a case with successful anterior primary repair and a literature review to provide more alternatives for effective management of these injuries.

## Supplementary Material

Surgical_Repair_GEJ_injury_rjab160
